# Surgical transitional care interventions and their outcomes: a scoping review

**DOI:** 10.1016/j.ijnsa.2025.100328

**Published:** 2025-04-08

**Authors:** G. Tobiano, W. Chaboyer, K. Turner, A.M. Eskes, B. Patel, J. Colquhoun, L. Ferronato, B.M. Gillespie

**Affiliations:** a NHMRC Centre of Research Excellence in Wiser Wound Care, Griffith University, Gold Coast Campus, 1 Parklands Dr, Southport, QLD Australia 4222; b Gold Coast University Hospital, Gold Coast Hospital & Health Service, 1 Hospital Blvd, Southport, QLD 4215, Australia; c School of Nursing and Midwifery, Griffith University, Gold Coast Campus, 1 Parklands Dr, Southport, QLD Australia 4222; d Griffith University, Gold Coast Campus, 1 Parklands Dr, Southport, QLD 4222, Australia; e Department of Surgery, Amsterdam UMC, Meibergdreef 9, 1105 AZ Amsterdam; f Princess Alexandra Hospital, 199 Ipswich Road, Woolloongabba, QLD Australia 4102; g Bond University, 14 University Dr, Robina QLD 4226

**Keywords:** Continuity of patient care, Hospital to home transition, Patient discharge, Postoperative care, Review, Surgical procedures, Operative, Transitional care

## Abstract

**Background:**

The few reviews available on surgical transitional care interventions focus on intervention effectiveness on readmissions, showing that transitional care interventions may reduce readmissions. More detailed guidance is needed for clinicians and researchers to adapt and implement these interventions and evaluate their effect.

**Objective:**

To identify, synthesise and map the evidence on surgical transitional care intervention components and surgical patient outcomes.

**Design:**

Scoping review.

**Methods:**

Medline, CINAHL and EMBASE were searched, followed by backward and forward citation searching. Two researchers independently screened titles and abstracts, and then full-texts. Data were extracted about study and intervention characteristics by one researcher, and checked for accuracy by a second researcher. To summarise the data, intervention components and outcome measures were categorised according to an existing list of transitional care intervention components and a core outcome set for perioperative patients, which were presented as tables, figures, and text.

**Results:**

Of 5176 articles found, 30 studies were included. Most studies focussed on cardiothoracic, general and orthopaedic surgery, and were primarily conducted in Asia and North America. Outcomes frequently measured were hospital readmissions, followed by health-related quality of life. Pre-discharge assessment, education and discharge planning, post-discharge telephone calls, and caregiver involvement were the most common intervention components. Generally, they demonstrated positive outcomes for hospital readmission and patient satisfaction.

**Conclusions:**

There is large focus on re-admission as an outcome measure, presenting an opportunity to explore a broader range of patient-centred and transition specific outcome measures. While common transitional care intervention components were uncovered, the evidence-base for each individual component is unclear. Gaps were found in surgical populations and settings, with most transitional care interventions focussing on cardiothoracic surgery across a limited geographic context, highlighting the opportunity to build the evidence-base for surgical transitional care interventions across a range of contexts.

**Review registration:**

The Open Science Framework (https://osf.io/kf2v7/).

**Contributions of the paper**

What is already known?

- Transitional care interventions, which support patients and caregivers from hospital to home, may reduce hospital re-admissions.
- Existing reviews on transitional care interventions for surgical patients tend to focus on intervention effectiveness. There is a need to provide guidance on how to prioritise intervention components and outcomes for these interventions.

What this paper adds

- The most common surgical transitional care intervention components were pre-discharge assessment, education and discharge planning, post-discharge telephone calls, and caregiver involvement.
- The most measured outcomes were hospital readmission and healthcare-related quality of life. We suggest that a wider range of outcomes could be measured, including outcomes specific to transitions in care.
- Most studies were conducted in Asia and North America, with most evidence available for cardiothoracic patients.

Alt-text: Unlabelled box

## 1 Background

One-in-seven patients who have undergone major surgery are readmitted to hospital within 30 days of discharge (Tsai et al., 2013). The risk of death doubles for people readmitted to hospital compared to those not readmitted, which are a financial burden for hospitals (Upadhyay et al., 2019; Shaw et al., 2020). Yet, one-in-five of these readmissions are potentially preventable (Brown et al., 2021), with 30-day readmission being significantly lower amongst patients who feel prepared (Martin et al., 2017). However, patients and their caregivers have reported not knowing what to expect after surgery and after they return home; necessary information to partner in the recovery process (Brooke et al., 2018). Because some patients lack the information and skills to manage their postoperative recovery at home (Jaensson et al., 2019), many access emergency services for concerns, regardless of how trivial (Kang et al., 2020). Ultimately, the transition home for surgical patients and their caregivers is a vulnerable time.

Surgical patients who receive transitional care interventions are 40 % less likely to be readmitted to hospital (Mao et al., 2022). Transitional care interventions are multi-component, starting in hospital and continuing after discharge, to support patients and their caregivers to manage recovery (Holland and Harris, 2007). Researchers have shown that the individual components of surgical transitional care interventions reduce readmissions. For example, discharge planning components reduced readmission by 12–23 %; patient education reduced readmission by 14–24 % and home visits by 4–8 % (Jones et al., 2016). Despite the individual effectiveness of each component, researchers purport that combining components into multi-faceted surgical transitional care interventions has the best impact on surgical patients (Laugaland et al., 2012).

Previous reviews on transitional care interventions generally do not focus on surgical patient populations (Laugaland et al., 2012; Allen et al., 2014; Mabire et al., 2016; Rasmussen et al., 2023; Tyler et al., 2023), with many focused specifically on older people (Laugaland et al., 2012; Allen et al., 2014; Mabire et al., 2016; Rasmussen et al., 2023; Hladkowicz et al., 2022). The reviews available on surgical transitional care interventions often examine their effect on hospital readmissions (Mao et al., 2022; Jones et al., 2016). However, clinicians and researchers planning to develop and test surgical transitional care interventions need to systematically prioritize a range of outcomes that are meaningful and relevant to those affected by the research. Additionally, these reviews tend to investigate the effect of surgical transitional care interventions as a whole and lack details about the individual components within the interventions (Mao et al., 2022; Jones et al., 2016). Clinicians and researchers selecting surgical transitional care intervention components require further guidance about the range of available components when adapting transitional care interventions to their setting. Further, there is limited understanding of the intervention components that are core versus those that are discretionary. Core components are those that must be implemented consistently to ensure intervention integrity and effectiveness. Whereas discretionary components are adaptable and can be modified to suit the context/specific situation. Thus, this scoping review aimed to identify, synthesise, and map the evidence on surgical transitional care intervention components and surgical patient outcomes.

## 2 Design

A scoping review was conducted as they focus on the breadth of the research literature (Arksey and O'Malley, 2005; Peters et al., 2015), and allows reviewers to summarise and synthesise literature, to determine research gaps and recommend directions for further studies (Davis et al., 2009; Tricco et al., 2016). This review followed the six methodological stages outlined by Arksey and O'Malley (Arksey and O'Malley, 2005) and further developed by Peter et al. (Peters and al., 2020). These stages include: (1) identifying the research question, (2) identifying relevant articles, (3) study selection, (4) charting the data, (5) collating, summarising and reporting the results, and (6) consultation. The review protocol was registered on the Open Science Framework (https://osf.io/kf2v7/).

### 2.1 Identifying the research question

The primary aim was to identify, synthesise, and map the evidence on surgical transitional care intervention components and their outcomes for surgical patients undergoing planned and emergency surgeries. Specifically, the review questions included:

- What are the core versus the discretionary components of transitional care interventions used for adult surgical patients?
- What outcomes are used to evaluate transitional care interventions for adult surgical patients?

This scoping review used the population, concept and context (PCC) approach (Peters et al., 2015) to develop the research questions (Table 1).

**Table 1:** Population, concept and context for this scoping review.

PCC elements	Components
POPULATION	Adult patients undergoing planned and emergency surgeries. Including both groups. Including both populations broadens the scope of evidence, ensuring that the review findings are relevant to a wider patient population. .
CONCEPT	Transitional care interventions, defined as multi-component interventions (≥ 2 components) that are a distinct element or activity as part of the larger intervention designed to contribute to specific outcomes. Each component contributes to the intervention's effectiveness. In this review, transitional care interventions span pre- and post-hospital settings; components may include support services and follow-up activities (Holland and Harris, 2007).
CONTEXT	Spans hospital and post-hospital settings where the patient/caregiver has responsibility for managing care.

### 2.2 Identifying relevant articles study selection

The computerised database search was developed with the help of a health librarian, and by referring to key articles in this field, which informed subject headings and search term identification (See Supplementary File 1). Searches were conducted in Medline, CINAHL and EMBASE. Next, back and forward citation searching was conducted. That is, reference lists of articles included in this review were hand-searched (backward search), and included articles were searched in the Scopus database to identify articles that had cited their work (forward search).

Prior to article selection, search results were exported to EndNote 20 where duplicates were removed, and then uploaded into the Covidence systematic review software (Covidence). Covidence was used to remove duplicates a second time, and then articles were screened independently by two researchers based on the inclusion and exclusion criteria (See Table 2). The researchers screened titles and abstracts, and then full texts. Any disagreements were resolved by discussion. Unresolved disagreements were adjudicated by a third researcher. For articles with unclear reporting components, authors were contacted via e-mail for clarification. The description of the article selection is presented in narrative and a flow diagram as per reporting guidelines (Tricco et al., 2018).

**Table 2:** Inclusion and exclusion criteria for articles.

Inclusion criteria	
Criteria:	Description and/or rationale:
Transitional care interventions targeted at adult surgical inpatients.	- Aligns with our phenomenon of interest.
- Transitional care interventions with ≥ 2 components.	- Aligns with our phenomenon of interest.
- ≥ 1 component delivered by nurse.	- Nurses have a key role in transitional care interventions (Mao et al., 2022).
- Reports outcomes.	- Aligns with our research questions.
- Original peer reviewed research and quality improvement projects.	- Captures broad range of transitional care interventions, not only those evaluated using randomised control trial methodologies (Mao et al., 2022; Jones et al., 2016).
- In English.	- Due to the abilities of team, and English being the most accessible language, universally spoken.
- Date range 2016–2023.	- In 2016, the WHO released their seminal report providing international recommendations for improving transitions of care (World Health Organization, 2016).

Exclusion criteria	
Criteria:	Description and/or rationale:
- Conducted in rehabilitation setting or care facilities, where post admitted surgical care patients receive 24-hour care from healthcare professionals.	- Represents a different care pathway.
- Included paediatric or other populations where the outcomes for adult surgical patients cannot be separated from other patient populations.	- These populations have a unique approach to transitional care interventions.

### 2.3 Charting the data

One researcher extracted data on a purpose-built form, which was pilot-tested on six articles. A second researcher checked its accuracy. For disagreements between the two researchers, a third researcher was available to adjudicate, but this was not required. Characteristics extracted included author/year/country, aim, study design, setting, sample, and surgery type. Data were also extracted about the intervention including whether components were core or discretionary and the foundation of the bundle (where reported).

### 2.4 Collating, summarising and reporting the results

Information in data extraction tables were summarised, using descriptive numerical summaries.

To identify patterns in the data we mapped the data in the following ways:

1. Interventions: We summarised the transitional care intervention components in a table using Rasmussen et al.’s (Rasmussan et al., 2021) list of surgical transitional care intervention components across the trajectory of care that included predischarge, bridging, and post-discharge intervals (e.g., patient assessment, education, physical exercise, counselling, caregiver involvement, handover, home visits, multidisciplinary care) as column labels. We included additional labels (e.g., an app, patient support groups, clinic visits, protocols for emergency department presentations and rehabilitation), that were inductively identified during analysis. We coded intervention components based on the authors’ descriptions of whether all components needed to be implemented (core) or whether the number of components could vary or be adapted to the context (discretionary).
2. Outcomes: We summarised patient outcomes, using a table with the outcomes as row labels. For the labels we used the ‘core outcome measures for perioperative patients’, which includes outcomes such as survival, post-operative complications (e.g., unplanned hospital readmission), resource use, and quality of life (Boney et al., 2022). We added a row for patient experience and costs, guided by the Triple Aim for Healthcare Framework (Berwick et al., 2008), which includes providing more effective care, a better patient experience and improved per capita costs. Additionally, we included labels identified inductively during analysis.

In scoping reviews, researchers are increasingly using creative data visualisation processes to show gaps, and ensure that findings are user-friendly for those using the review findings (South and Rodgers, 2023). There is a plethora of data visualization tools that can be used in scoping reviews. In this review a figure was used to map our patterns. Intervention and study characteristic patterns were row labels and outcomes were column labels. Studies were mapped to each cell and varying colours and numbers were used to represent the number of studies reporting each intervention, study characteristic or positive outcome pattern in the figure.

### 2.5 Consultation

A health consumer with lived experience of surgery and a clinical nurse and surgeon who engage with surgical patients transitioning from hospital to post-hospital settings were engaged in this review. They interpreted the data extraction and patterns found, and provided input into the creative data visualisation of research patterns and gaps. Their interpretations were used to guide discussion ideas, and the manuscript overall. Health consumer engagement is reported as per the GRIPP2 reporting checklist (Staniszewska et al., 2017) (See Supplementary File 2).

## 3 Findings

**Fig. 1:**
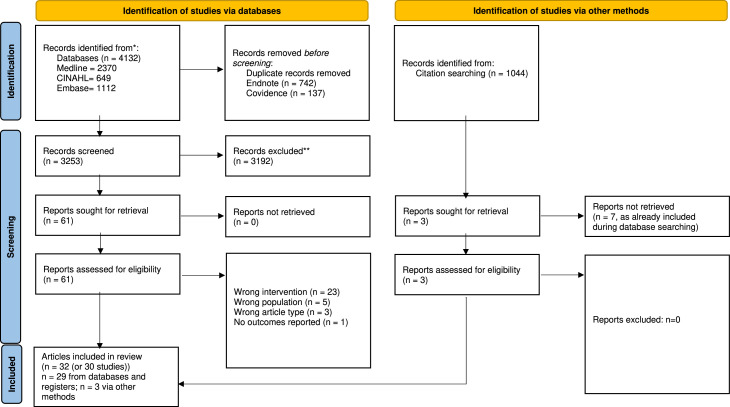
PRISMA flowchart (Tsai et al., 2013). 1. Page, M.J., et al., *The PRISMA 2020 statement: an updated guideline for reporting systematic reviews.* BMJ, 2021. **372**: p. n71.

The search was conducted in August 2023. In total, database searching and backwards and forward citation searching yielded 5176 articles (Fig. 1). A total of 32 articles were included in this review; comprising 30 studies. The articles by Robertson et al. and Liu at al. (Robertson et al., 2018; Liu et al., 2019) and Zhang et al. and Zhang et al. (Zhang et al., 2020; Zhang et al., 2021) report on the same two studies and interventions, however each published article reports different outcomes. Thus both Robertson et al.’s and Liu at al.’s (Robertson et al., 2018; Liu et al., 2019) articles and Zhang et al.’s and Zhang et al.’s (Zhang et al., 2020; Zhang et al., 2021) articles were counted as one study each in the findings.

## 4 Summary of study characteristics

See Supplementary File 3 for characteristics of the 30 studies. Study designs included 8/30 (27 %) RCTs (Zhang et al., 2020; Zhang et al., 2021; Coskun and Duygulu, 2022; Grahn et al., 2019; Hu et al., 2020; Tian et al., 2023; Tseng et al., 2021; Tseng et al., 2016; Zhou et al., 2020), 6/30 (20 %) cohort studies (Ahmadi et al., 2021; Borregaard et al., 2019; Lee et al., 2021; Shargall et al., 2016; Wang et al., 2023; Zuckerman et al., 2020), 7/30 (23 %) quality improvement projects (Robertson et al., 2018; Liu et al., 2019; Fisher et al., 2018; Fitz et al., 2020; Iseler et al., 2018; Koeckert et al., 2017; Mitchell, 2022; Pelt et al., 2018), 7/30 (23 %) quasi-experimental studies (Akbari and Celik, 2018; Lee, 2017; Xu et al., 2021; Yang et al., 2023; Aicher et al., 2019; Koçan and Gürsoy, 2023; Li et al., 2020), 1/30 (3 %) pilot interventional study and 1/30 (3 %) observational study (Du et al., 2021; Weintraub et al., 2018). The median sample size was 136 patients (IQR 60.5, 696.0) (range 6–1827); 1/30 (3 %) study did not report sample size. Two studies (7 %) also included caregivers in addition to patients, ranging from 125 to 152 caregivers. Surgical transitional care interventions were most frequently designed for cardiothoracic surgery (12/30 (40 %)) (Coskun and Duygulu, 2022; Ahmadi et al., 2021; Borregaard et al., 2019; Lee et al., 2021; Shargall et al., 2016; Fitz et al., 2020; Iseler et al., 2018; Koeckert et al., 2017; Akbari and Celik, 2018; Lee, 2017; Aicher et al., 2019; Weintraub et al., 2018), general surgery (7/30 (23 %)) (Zhang et al., 2020; Zhang et al., 2021; Grahn et al., 2019; Wang et al., 2023; Fisher et al., 2018; Du et al., 2021) inclusive of breast surgery (Zhou et al., 2020; Koçan and Gürsoy, 2023), and orthopaedic surgery (6/30 (20 %)) (Tseng et al., 2021; Tseng et al., 2016; Zuckerman et al., 2020; Mitchell, 2022; Pelt et al., 2018; Xu et al., 2021), with the rest for various types of surgery. Half (15/30 (50 %)) of the included studies were conducted in North America (Robertson et al., 2018; Liu et al., 2019; Grahn et al., 2019; Ahmadi et al., 2021; Shargall et al., 2016; Zuckerman et al., 2020; Fisher et al., 2018; Fitz et al., 2020; Iseler et al., 2018; Koeckert et al., 2017; Mitchell, 2022; Pelt et al., 2018; Lee, 2017; Aicher et al., 2019; Du et al., 2021; Weintraub et al., 2018) and nearly half in Asia (14/30 (47 %)) (Zhang et al., 2020; Zhang et al., 2021; Coskun and Duygulu, 2022; Hu et al., 2020; Tian et al., 2023; Tseng et al., 2021; Tseng et al., 2016; Zhou et al., 2020; Lee et al., 2021; Wang et al., 2023; Akbari and Celik, 2018; Xu et al., 2021; Yang et al., 2023; Koçan and Gürsoy, 2023; Li et al., 2020). For studies that provided details about the hospital setting, many were academic hospitals (12/30 (40 %)) (Robertson et al., 2018; Liu et al., 2019; Coskun and Duygulu, 2022; Grahn et al., 2019; Ahmadi et al., 2021; Borregaard et al., 2019; Lee et al., 2021; Shargall et al., 2016; Fisher et al., 2018; Fitz et al., 2020; Yang et al., 2023; Aicher et al., 2019; Koçan and Gürsoy, 2023), and tertiary level hospitals (9/30 (30 %)) (Hu et al., 2020; Tian et al., 2023; Ahmadi et al., 2021; Wang et al., 2023; Fisher et al., 2018; Mitchell, 2022; Pelt et al., 2018; Xu et al., 2021; Du et al., 2021).

## 5 Interventions

Transitional care interventions were implemented from 2016 onwards. In all but one study (Fisher et al., 2018), all intervention components were implemented as core components. Surgical transitional care interventions had a median of seven components (range: 3–12) (see Table 3). The most frequently reported components pre-discharge were education, assessment, and discharge planning. Bridging components, which are intervention components that facilitate/arrange post-discharge care, were usually handover/coordination of care and scheduling follow-up. Post-discharge, telephone follow-up/telemedicine, having healthcare professionals available for patients to contact when needed, and clinic visits were most common. Caregiver involvement occurred in 19/30 (63 %) studies, across both pre- and post- discharge components.

**Table 3:** Intervention components.

Components found inductively included having a dedicated staff member provide the transitional care intervention and rehabilitation pre-discharge. In the bridging phase, electronic systems to centralise patient data and scheduling of routine follow-up were inductive components found. For post-discharge, inductive components were follow-up using an app, patient support groups, clinic visits, protocols for emergency department presentations and rehabilitation. Finally, we moved caregiver involvement from the pre-discharge phase to its own column, as we found that this occurred in many intervention components, across phases (i.e. pre-discharge and post-discharge).

The interventions for all but 8/30 (27 %) studies were developed based on a literature review (Zhang et al., 2020; Zhang et al., 2021; Coskun and Duygulu, 2022; Grahn et al., 2019; Hu et al., 2020; Tian et al., 2023; Tseng et al., 2021; Tseng et al., 2016; Zhou et al., 2020; Ahmadi et al., 2021; Borregaard et al., 2019; Lee et al., 2021; Wang et al., 2023; Zuckerman et al., 2020; Fisher et al., 2018; Fitz et al., 2020; Iseler et al., 2018; Akbari and Celik, 2018; Lee, 2017; Yang et al., 2023; Koçan and Gürsoy, 2023; Li et al., 2020; Weintraub et al., 2018) or stakeholder input (13/30 studies (43 %)) (Zhang et al., 2020; Zhang et al., 2021; Grahn et al., 2019; Hu et al., 2020; Zhou et al., 2020; Zuckerman et al., 2020; Fisher et al., 2018; Fitz et al., 2020; Koeckert et al., 2017; Pelt et al., 2018; Akbari and Celik, 2018; Xu et al., 2021; Koçan and Gürsoy, 2023; Du et al., 2021). Additional intervention data extraction is available in Supplementary File 4.

## 6 Outcomes

Based on the core outcome set used, researchers tended to focus on resource use and longer-term recovery after surgery (Table 4). For resource use, unplanned hospital readmission was the most frequent outcome reported in 19/30 (63 %) studies, with half reporting positive results (Robertson et al., 2018; Liu et al., 2019; Coskun and Duygulu, 2022; Hu et al., 2020; Ahmadi et al., 2021; Borregaard et al., 2019; Iseler et al., 2018; Pelt et al., 2018; Lee, 2017; Aicher et al., 2019). For longer-term recovery after surgery, health related quality of life was reported in 9/30 (30 %) studies, with positive results found in 6/30 (20 %) studies (Coskun and Duygulu, 2022; Tian et al., 2023; Zhou et al., 2020; Wang et al., 2023; Akbari and Celik, 2018; Koçan and Gürsoy, 2023).

**Table 4:** Outcomes measured.

Outcomes	# studies effective	References	# studies not effective	References	# studies mixed results	References	Can't tell or results not reported for the outcome	References
**Mortality/ survival:**								
Mortality	1	(Ahmadi et al., 2021)	3	(Tseng et al., 2021; Borregaard et al., 2019; Shargall et al., 2016)	0	N/A	1	(Koeckert et al., 2017)
Overall long-term survival	0	N/A	0	N/A	0	N/A	0	N/A
**Perioperative complications:**								
Major (serious) postoperative complications and adverse events	1	(Li et al., 2020)	1	(Tseng et al., 2021)	3	(Zhang et al., 2020; Zhang et al., 2021^a^; Wang et al., 2023^b^; Koçan and Gürsoy, 2023_]_^a^	0	N/A
Complications and adverse events causing permanent disability or harm	0	N/A	0	N/A	0	N/A	0	N/A
**Resource use:**								
Total number of days spent in hospital for the operation	5	(Robertson et al., 2018; Liu et al., 2019; Ahmadi et al., 2021; Shargall et al., 2016; Zuckerman et al., 2020; Aicher et al., 2019)	2	(Grahn et al., 2019; Tseng et al., 2021)	0	N/A	1	(Koeckert et al., 2017)
Unplanned hospital readmission	9	(Robertson et al., 2018; Liu et al., 2019; Coskun and Duygulu, 2022; Hu et al., 2020; Ahmadi et al., 2021; Borregaard et al., 2019; Iseler et al., 2018; Pelt et al., 2018; Lee, 2017; Aicher et al., 2019)	8	(Grahn et al., 2019; Tian et al., 2023; Lee et al., 2021; Wang et al., 2023; Zuckerman et al., 2020; Fitz et al., 2020; Mitchell, 2022; Weintraub et al., 2018)	1	(Shargall et al., 2016) d	1	(Koeckert et al., 2017)
Costs^e^	3	(Robertson et al., 2018; Liu et al., 2019) (Shargall et al., 2016) (Tian et al., 2023)	2	(Grahn et al., 2019; Weintraub et al., 2018)	0	N/A	1	(Ahmadi et al., 2021)
**Short-term recovery after surgery:**								
Discharge destination from hospital	1	(Wang et al., 2023)	0	N/A	0	N/A	0	N/A
**Longer-term recovery after surgery:**								
Overall health-related quality of life	6	(Coskun and Duygulu, 2022; Tian et al., 2023; Zhou et al., 2020; Wang et al., 2023; Akbari and Celik, 2018; Koçan and Gürsoy, 2023)	1	(Weintraub et al., 2018)	1^c^	(Zhang et al., 2020; Zhang et al., 2021) a	1	(Tseng et al., 2021)
Functional status^f^	4	(Zhang et al., 2020; Zhang et al., 2021; Coskun and Duygulu, 2022; Yang et al., 2023; Li et al., 2020)	3	(Tian et al., 2023; Tseng et al., 2021; Weintraub et al., 2018)	0	N/A	0	N/A
**Short-term recovery after surgery:**								
Pain	1	(Yang et al., 2023)	2	(Tian et al., 2023; Zhou et al., 2020)	0	N/A	0	N/A
Nausea with or without vomiting	0	N/A	0	N/A	0	N/A	0	N/A
Mental, emotional, and psychological wellbeing	1	(Zhang et al., 2020; Zhang et al., 2021)	0	N/A	3^c^	(Koçan and Gürsoy, 2023; Tseng et al., 2021) a	0	N/A
**Overall success/ failure of surgery:**								
Patient satisfaction with their operation, willingness (with hindsight) to choose the same again, or both	0	N/A	0	N/A	0	N/A	0	N/A
**Patient reported experience and satisfaction:**								
Patient experience^e^	1	(Du et al., 2021)	0	N/A	0	N/A	0	N/A
Patient satisfaction with healthcare professional behaviour, care processes or the intervention^f^^,^^g^	8	(Robertson et al., 2018; Liu et al., 2019; Hu et al., 2020; Shargall et al., 2016; Wang et al., 2023; Zuckerman et al., 2020; Fisher et al., 2018; Mitchell, 2022; Li et al., 2020)	1	(Grahn et al., 2019)	0	N/A	0	N/A
**Other:**								
Care transition quality^f^	5	(Hu et al., 2020; Wang et al., 2023; Fisher et al., 2018; Xu et al., 2021; Du et al., 2021)	1	(Weintraub et al., 2018)	0	N/A	0	N/A
Discharge readiness^f^	3	(Hu et al., 2020; Zuckerman et al., 2020; Mitchell, 2022)	0	N/A	1	(Wang et al., 2023)	1	(Koeckert et al., 2017)

a Dependent on time point of data collection.

b Dependent of type of postoperative complication, for example, in Wang et al. (2023), there was significantly less wound-related issues in the intervention group, whereas no significant difference was found in electrolyte disorders between groups (Wang et al., 2023).

c In Zhang et al. and Zhang et al. (Zhang et al., 2020; Zhang et al., 2021) outcomes for caregivers were also measured. There were mixed results for caregiver “overall health-related quality of life” and “mental, emotional, and psychological wellbeing”, depending on time point of data collection.

d Dependent on which sub group was analysed.

e Added by the research team to align with Triple Aim for Healthcare Framework (Berwick et al., 2008).

f Outcome found inductively during analysis.

g Hu et al. (Hu et al., 2020) and Fisher et al. (Fisher et al., 2018) measured satisfaction with the intervention.

Outcomes that aligned with the Triple Aim for Healthcare Framework (Berwick et al., 2008), were measured infrequently. Costs were measured in six studies, with three studies showing positive outcomes (Robertson et al., 2018; Liu et al., 2019; Shargall et al., 2016; Tian et al., 2023; Grahn et al., 2019; Weintraub et al., 2018; Ahmadi et al., 2021). While patient experience was measured in one study that had positive results (Du et al., 2021).

Outcomes found inductively that were not part of the core outcome set used, included patient satisfaction, care transition quality, discharge readiness and functional status. Patient satisfaction with healthcare professionals and care processes and the intervention, was measured in 9/30 (30 %) studies, with 8/30 (27 %) studies showing positive results (Robertson et al., 2018; Liu et al., 2019; Hu et al., 2020; Shargall et al., 2016; Wang et al., 2023; Zuckerman et al., 2020; Fisher et al., 2018; Mitchell, 2022; Li et al., 2020). We also identified two other outcomes that were frequently measured. In 6/30 (20 %) studies, care transition quality was measured, with 5/30 (17 %) studies showing positive results (Hu et al., 2020; Wang et al., 2023; Fisher et al., 2018; Xu et al., 2021; Du et al., 2021). Measures used included the Care Transition Composite (CTM-3) (Du et al., 2021; Weintraub et al., 2018), Care Transition Measure-15 (CTM-15) (Hu et al., 2020; Wang et al., 2023), Care Transition Measure – Chinese (CTM-C), and a survey designed by the research team (Fisher et al., 2018). Additionally, discharge readiness was measured in 4/30 (13 %) studies, with 3/30 (10 %) studies showing positive results (Hu et al., 2020; Zuckerman et al., 2020; Mitchell, 2022). Researchers used the Readiness for Discharge Scale (RHDS) (Hu et al., 2020; Wang et al., 2023; Weiss and Piacentine, 2006) and a 0–10 scale for patients to rate readiness for discharge (Zuckerman et al., 2020; Mitchell, 2022). Finally, positive outcomes were found for functional status in 4/30 (13 %) studies (Zhang et al., 2020; Zhang et al., 2021; Coskun and Duygulu, 2022; Yang et al., 2023; Li et al., 2020).

## 7 Creative data visualisation of research patterns and gaps

**Fig. 2:**
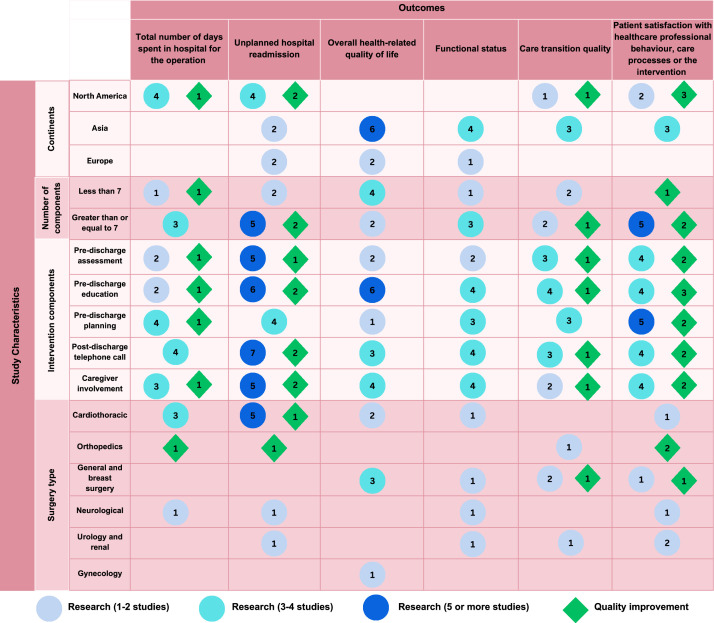
Creative data visualisation.

*Note:* only studies that reported positive outcomes are included in the visualisation; for “number of components” the cut-off of 7 components was selected based on the median number of components found in this review; the most frequent intervention components found in Table 4 are presented in the visualisation; *n* = 9 studies included in this review had positive outcomes for unplanned hospital re-admission, however *n* = 10 studies are shown for “continents” because Coskun et al. (Zhang et al., 2020) was conducted in Turkey which is considered by an Asian and European country.

Fig. 2 highlights that different ‘positive’ outcomes found across countries, for example in North American countries researchers found positive outcomes for re-admission and days spent in hospital, while in Asian countries health-related quality of life measures was reported more often. In general, interventions with greater than or equal to seven intervention components tended to show more positive outcomes, but more evidence is needed for outcomes like health-related quality of life and care transition quality. The most common intervention components (pre-discharge assessment, pre-discharge education, pre-discharge planning, post-discharge telephone call and caregiver involvement) tended to show positive effects on unplanned hospital readmission and patient satisfaction, however these results are largely from quality improvement studies. The most positive evidence for unplanned readmissions was for cardiothoracic surgery transitional care interventions, while the evidence for other outcomes across surgeries was sparse.

## 8 Discussion

In summary, using the perioperative core outcome set, unplanned hospital readmissions were most frequently measured. Outcomes identified inductively like care transition quality and discharge readiness, were measured often. Common intervention components were pre-discharge assessment, education and discharge planning, post-discharge telephone calls, and caregiver involvement, which may influence hospital readmission and patient satisfaction, but further evidence is required. Finally, the vast majority of studies were conducted in Asia and North America, with the most abundant evidence available for cardiothoracic patients.

Our review highlights the tension between focusing on hospital utilisation versus a more holistic, patient-centred range of outcomes. It was unsurprising that re-admissions were measured in nearly two-thirds of largely North American studies included in this review, given that the United States receives financial penalties for this traditionally measured outcome (James et al., 2023). However, heavily focussing on re-admission data can overshadow the value of measuring patient-centred outcomes, like quality of life, which was measured in only one-third of studies in this review. Core outcome sets for transitions of care for children with medical complexity and people discharged from inpatient mental health facilities, which were developed with patients/parents and healthcare professionals, both list quality of life and re-admissions as important outcomes (Haspels et al., 2023; Tyler et al., 2020). This may reflect the diverse views of patients/parents compared to healthcare professionals, suggesting a blend of resource utilisation and patient-centred outcomes is viewed as essential to measure. Ultimately, re-admission data is needed to understand patients’ unmet needs and the cost implications for hospitals (e.g. beds and theatre costs) (Reeves et al., 2021), but this must be balanced with measurement of outcomes that reflect patients’ wide-ranging experiences of transitions of care.

The need for standardised and transition-specific outcomes for transitional care interventions is underscored by our review findings. Care transition quality and discharge readiness were concepts measured in a few studies included in this review; measures absent in the ‘core outcome measures for perioperative patients’ that we used to guide analysis. Questionnaires used to measure these concepts like the ‘Care Transition Measure’ and ‘Readiness for Discharge Scale’ have been validated for use in the context of transitional care (Reeves et al., 2021; Feldbusch et al., 2024). Additionally, they may be important outcomes for patients, with the ‘Care Transition Measure’ developed using patient-centred methodology (Reeves et al., 2021), and the concept of discharge readiness being essential to achieving patient-centred care in surgical settings (Leonardsen et al., 2024). There are calls to develop a core outcome set for transitions in care, which must include outcomes prioritised by patients, and be validated for use in the transitional care context (Tyler et al., 2023; Reeves et al., 2021), it may be that ‘Care Transition Measure’ and ‘Readiness for Discharge Scale’ are an appropriate fit.

Consistent with previous work, we uncovered that components like follow-up phone calls and patient education were commonly included in transitional care interventions; however, there were evidence gaps for these individual components. In a systematic review of the effect of transitional care interventions on re-admissions, patient education reduced readmission by 14 %, but there was minimal evidence for follow-up phone calls (Jones et al., 2016). When developing complex interventions like transitional care interventions, each individual intervention component must be based on high-quality evidence, as components act in an additive way (Cochrane). Another consideration is that highly effective intervention components are of little value if they are not feasible to implement. In other research, patients found follow-up phone calls acceptable (Arnaert et al., 2022), and nurses believed they were non-burdensome, even when patients were provided with the nurse's phone number to call if required (Dahlberg et al., 2019). For example, in a study of 494 surgical patients, only 17 % initiated a follow-up phone call to a nurse (Dahlberg et al., 2019). Patients have reported feeling reassured knowing a nurse is available by phone if needed (Arnaert et al., 2022). Additionally, components like discharge education have been viewed by surgical patients as useful and satisfactory, and accessed by patients more than once (Kang et al., 2022). It appears more head-to-head trials are needed to distinguish which intervention components work best, with parallel feasibility and health economic assessments, although it is clear these would be resource intensive trials.

We found gaps in surgical transitional care intervention research in terms of surgical populations and settings. For surgical populations, most evidence for transitional care interventions was for cardiothoracic patients; investigation of outcomes for a broader range of both elective and emergency surgeries may be required. For example, the complexity of re-admissions and associated costs, varies across surgeries effecting this outcome (Parry et al., 2021). Additionally, most research was conducted in North America and Asia; it is often impossible to separate interventions from the context in which they are delivered (Shiell et al., 2008), suggesting the effectiveness of transitional care interventions may differ across countries. For example, while there were few studies conducted in Europe, there is a large focus on elective surgery using enhanced recovery after surgery pathways to shorten length of stay in this context, which could influence transitional care intervention outcomes. Overall, more work is needed to determine which contextually-specific transitional care interventions work best, for which surgeries, in which settings.

## 9 Strengths and limitations

First, we developed an exhaustive search strategy, with expert input from a health librarian, but recognise that some studies may be missed. Second, we deviated from our published protocol, deciding to show patterns and gaps through creative data visualisation, rather than the PAGER framework (Bradbury-Jones et al., 2022). Scoping reviews allow for methods to evolve as the review progresses. We believe this decision strengthened our review by providing a clear picture for readers of where the evidence exists. Third, both research and quality improvement studies were included. While quality improvement studies may be less rigorous, they offer valuable insights that allow researchers to explore the mechanisms behind successful quality improvement strategies and provide information that may guide future rigorous trials. In line with scoping review methodology (Arksey and O'Malley, 2005), we did not undertake quality appraisal on any studies in this review, which may limit the usability of our findings.

## 10 Conclusion

Our review highlights that hospital re-admission is the most frequent outcome measured when evaluating surgical transitional care interventions. However, a more holistic, patient-centred and transition-specific exploration of outcomes could benefit the field. This review shows that common multi-component approaches to surgical transitional care interventions include patient assessment, education, and planning in the hospital, a phone call after discharge, and caregiver involvement throughout the transition. However, the evidence for these individual outcomes was inconclusive and primarily based on quality improvement studies. Overall, the results should be interpreted with caution, given that the research was mainly conducted in two continents, and with cardiothoracic patients. To advance the surgical transitional care intervention research agenda, more research is needed to explore transitional care intervention effectiveness across various surgeries and settings, to ensure these interventions are evidence-based and contextually appropriate.

## Funding

GT salary funded by the NHMRC Centre of Research Excellence in Wiser Wound Care (APP1196436). Open access publishing facilitated by Griffith University, as part of the Elsevier - Griffith University agreement via the Council of Australian University Librarians.

## CRediT authorship contribution statement

**G. Tobiano:** Writing – review & editing, Writing – original draft, Visualization, Validation, Project administration, Methodology, Investigation, Formal analysis, Data curation, Conceptualization. **W. Chaboyer:** Writing – review & editing, Visualization, Supervision, Resources, Methodology, Investigation, Formal analysis, Conceptualization. **K. Turner:** Writing – review & editing, Visualization, Validation, Project administration, Investigation, Formal analysis, Data curation. **A.M. Eskes:** Writing – review & editing, Visualization, Validation, Investigation, Formal analysis. **B. Patel:** Writing – review & editing, Visualization, Validation, Investigation, Formal analysis. **J. Colquhoun:** Writing – review & editing, Visualization, Validation, Formal analysis. **L. Ferronato:** Writing – review & editing, Writing – original draft, Validation, Formal analysis. **B.M. Gillespie:** Writing – review & editing, Visualization, Validation, Supervision, Resources, Methodology, Formal analysis, Conceptualization.

## Declaration of competing interest

The authors declare that they have no known competing financial interests or personal relationships that could have appeared to influence the work reported in this paper.

## Appendix Supplementary materials

Image, application 1

Image, application 2

Image, application 3

Image, application 4
