# Supplementary material for: Surgical transitional care interventions and their outcomes: a scoping review
Source: Int J Nurs Stud Adv. 2025 Apr 8;8:100328. doi: 10.1016/j.ijnsa.2025.100328 (PMC12136900; doi:10.1016/j.ijnsa.2025.100328)
Supplement: Supplementary file 1 [file mmc1.docx]

**Supplementary File 1.**

Search strategy in Medline (EBSCOhost)

| **#** | **Query** |
| --- | --- |
| S9 | Limiters - Date of Publication  Limiters - Age |
| S8 | S3 AND S7 |
| S7 | S4 OR S5 OR S6 |
| S6 | (MH "Transitional Care") |
| S5 | ((TI Transition* OR AB Transition*) N2 (TI care OR AB care)) |
| S4 | (((TI Transition* OR AB Transition*) OR (TI discharge OR AB discharge) OR (TI postdischarge OR AB postdischarge) OR (TI post-discharge OR AB post-discharge)) N2 ((TI intervention OR AB intervention) OR (TI program OR AB program) OR (TI service OR AB service) OR (TI pathway OR AB pathway) OR (TI plan* OR AB plan*))) |
| S3 | S1 OR S2 |
| S2 | AB ( perioperative or peri-operative or pre-operative or preoperative or post-operative or postoperative or surg* or operat* ) OR TI ( perioperative or peri-operative or pre-operative or preoperative or post-operative or postoperative or surg* or operat* ) |
| S1 | (MH "Surgical Procedures, Operative+") |

Search strategy in CINAHL (EBSCOhost)

| # | **Query** |
| --- | --- |
| S13 | Limiters - Date of Publication |
| S12 | S3 AND S11 |
| S11 | S6 OR S9 OR S10 |
| S10 | (MH "Transitional Care") |
| S9 | S7 N2 S8 |
| S8 | TI care OR AB care |
| S7 | TI Transition* OR AB Transition* |
| S6 | S4 N2 S5 |
| S5 | TI ( intervention or program or service or pathway or plan* ) OR AB ( intervention or program or service or pathway or plan* ) |
| S4 | TI ( Transition* or discharge or postdischarge or post-discharge ) OR AB ( Transition* or discharge or postdischarge or post-discharge ) |
| S3 | S1 OR S2 |
| S2 | TI ( perioperative OR peri-operative OR pre-operative OR preoperative OR post-operative OR postoperative OR surg* OR operat* ) OR AB ( perioperative OR peri-operative OR pre-operative OR preoperative OR post-operative OR postoperative OR surg* OR operat* ) |
| S1 | (MH "Surgery, Operative+") |

Search strategy in Embase

| # | **Query** |
| --- | --- |
| S17 | Limiters - Date of Publication |
| S16 | S5 AND S15 |
| S15 | S1 AND S14 |
| S14 | S6 OR S7 OR S8 OR S9 OR S10 OR S11 OR S12 OR S13 |
| S13 | 'operat*':ab,ti |
| S12 | 'surg*':ab,ti |
| S11 | 'postoperative':ab,ti |
| S10 | 'post operative':ab,ti |
| S9 | 'preoperative':ab,ti |
| S8 | 'pre operative':ab,ti |
| S7 | 'peri operative':ab,ti |
| S6 | perioperative:ab,ti |
| S5 | S2 OR S3 OR S4 |
| S4 | transition* NEAR/2 care |
| S3 | (transition* OR discharge OR postdischarge OR 'post discharge') NEAR/2 (intervention OR program OR service OR pathway OR plan) |
| S2 | 'transitional care'/exp |
| S1 | 'surgery'/exp |
