# Supplementary material for: Surgical transitional care interventions and their outcomes: a scoping review
Source: Int J Nurs Stud Adv. 2025 Apr 8;8:100328. doi: 10.1016/j.ijnsa.2025.100328 (PMC12136900; doi:10.1016/j.ijnsa.2025.100328)
Supplement: Supplementary file 3 [file mmc3.docx]

**Supplementary File 3.** Study characteristics (N=30 studies)

| Author, year, country | Aim | Study design and setting | Sample and surgery type |
| --- | --- | --- | --- |
| Ahmadi et al., 2021  Canada | Assess novel integrated patient-centred, hospital-based multidisciplinary community program on post-discharge outcomes | **Design:** Retrospective cohort, including propensity score matching  **Setting:** Tertiary care academic institution | **n =** 1288 patients (692 intervention and 596 control^a^)  **Surgery type:** Lung resection |
| Aicher, Hanlon, Rosenberger, Toursavadkohi, & Crawford, 2019  USA | Evaluate the implementation and efficacy of system designed to reduce average length of stay | **Design:** Pretest-posttest  **Setting:** Medical-surgical telemetry units at university medical centre | **n =** 1697 patients (631 pre intervention and 1066 post intervention)  **Surgery type:** Vascular, including aortic, ruptured aortic aneurysm, lower extremity amputation, and lower extremity open surgery |
| Akbari & Celik, 2018  Iran | Investigate discharge training and post-discharge counselling on quality of life | **Design:** Quasi-experimental; pretest-posttest, with control group  **Setting:** Heart hospital | **n =** 100 patients (50 intervention and 50 control)  **Surgery type:** Coronary artery bypass graft |
| Borregaard et al., 2019  Denmark | Investigate early, individualised and intensified follow-up intervention on first, unplanned cardiac hospital readmission and all-cause mortality | **Design:** Prospective cohort, with a propensity matched historical control group  **Setting:** University hospital | **n =** 1288 patients (308 intervention and 980 control)  **Surgery type:** Open heart valve |
| Coskun & Duygulu, 2022  Turkey | Evaluate nurse led transitional care model on functional autonomy, quality of life, readmission, and rehospitalization rates | **Design:** RCT  **Setting:** One cardiovascular surgery clinic and a university hospital | **n =** 66 patients (33 intervention and 33 control)  **Surgery type**: Open heart |
| Du et al., 2021  USA | Document the adaptation of the Project RED intervention for surgery | **Design:** Pilot interventional, with matched control group  **Setting:** Veterans Affairs tertiary hospital | **n =** 45 patients (21 intervention and 24 comparison group)  **Surgery type:** Colectomy |
| Fisher et al., 2018  USA | Detail adaptive process for developing surgery-specific protocol to increase patient satisfaction | **Design:** Quality improvement project (posttest only)  **Settings:** Subspecialty surgical services of colorectal surgery and surgical oncology at a tertiary care, academic medical hospital | **n =** 30 patients  **Surgery types:** Complex abdominal: pancreatectomy, new ostomy patients, patients experiencing postoperative complications, or discharged with a drain in place. Extended to include: bowel perforations and emergent bowel resections, gastrectomy, tumour debulking with hyperthermic intraperitoneal chemotherapy |
| Fitz, Diegel-Vacek, & Mahoney, 2020  USA | Develop and implement evidence-based discharge bundle to standardize discharge planning process and reduce readmissions | **Design:** Quality improvement project (intervention group compared to retrospective and prospective chart audited data)  **Setting:** Academic medical centre | **Sample size:** Not reported  **Surgery type:** Lung transplantation |
| Grahn et al., 2019  USA | Determine if compliance to a program can decrease readmissions and prevent acute kidney injury | **Design:** Single-blinded RCT  **Settings:** One university hospital and two community hospitals | **n =** 100 patients (49 intervention and 51 control)  **Surgery type:** Ileostomy |
| Hu et al., 2020  China | Evaluate transitional care program in improving discharge readiness, transitional care quality, health services utilization and patient satisfaction | **Design:** RCT  **Setting:** General tertiary level hospital | **n =** 198 patients (100 intervention and 98 control)  **Surgery type:** Kidney transplantation |
| Iseler, Fox, & Wierenga, 2018  USA | Evaluate effectiveness and feasibility of transitional care model pilot program | **Design:** Quality improvement project (intervention group compared to retrospective and prospective chart audited data)  **Setting:** Large, urban, healthcare system | **n =** 10 patients  **Surgery type:** Left ventricular assist device implantation |
| Koçan & Gürsoy, 2023  Turkey | Determine effects of breast care training and follow-up on body image, anxiety, and quality of life | **Design:** Pretest-posttest design  **Setting:** University hospital | **n =** 34 patients  **Surgery type:** Modified radical mastectomy or breast-conserving |
| Koeckert et al., 2017  USA | Implement and evaluate effect of initiative on unplanned hospital readmission and readmission length of stay | **Design:** Quality improvement project (posttest only)  **Setting:** Department of cardiothoracic surgery | **n =** 345 patients  **Surgery type:** Cardiac; open and transcatheter valve |
| Lee, 2017  USA | Examine transitional care intervention on readmission rates | **Design:** Quasi-experimental; retrospective group (baseline usual care) and prospective group that received the intervention (posttest only)  **Setting:** Outpatient medical centre | **n =** 43 patients (19 pre-intervention and 24 post-intervention)  **Surgery type:** Cardiac transplant |
| Lee, Kang, Kim, & Chu, 2021  Korea | Compare effects of different discharge education approaches on clinical outcomes | **Design:** Retrospective cohort  **Setting:** University affiliated hospital | **n =** 136 patients; 3 groups: 25 usual care, 66 nurse led program, and 45 nurse led program + post-discharge education  **Surgery type:** Heart transplant |
| Li, Ma, & Wang, 2020  China | Establish transitional care group to improve self-care ability and quality of life | **Design:** Pretest-posttest  **Setting:** Urology department in a hospital | **n =** 100 patients  **Surgery type:** Kidney transplantation |
| Mitchell, 2022  USA | Determine impact of Reengineered Discharge Toolkit on patient knowledge for self-management, satisfaction with discharge process, discharge readiness, discharge time, and readmission | **Design:** Quality improvement project (posttest only)  **Setting:** One orthopaedic unit at a tertiary hospital in urban city | **n =** 30 patients  **Surgery type:** Hip or knee joint replacement or revision |
| Pelt et al., 2018  USA | Assess pathway on discharge disposition, readmissions and reoperations | **Design:** Quality improvement project (intervention group compared with matched case control group)  **Setting:** Tertiary referral centre | **n =** 927 patients (465 pre-implementation and 462 post- implementation)  **Surgery type:** Total joint arthroplasty of hip or knee |
| Liu et al., 2019; Robertson et al., 2018  USA | To decrease length of stay and readmissions and improve discharge efficiency using a transitional care program and perform a cost-benefit analysis | **Design:** Quality improvement project (cost benefit analysis and intervention group compared with matched control group)  **Setting:** Department of neurosurgery, at one large, urban, quaternary care, academic, referral hospital project | **n =** 832 patients (416 cases and 416 control)^b^  **Surgery type:** Cranial or spinal neurosurgery |
| Shargall et al., 2016  Canada | Evaluate integrated comprehensive care program on length of stay, readmission, emergency room visits, mortality and costs | **Design:** Retrospective cohort  **Setting:** Department of surgery at a hospital and academic health science centre | **n =** 686 patients (331 intervention and 355 control)  **Surgery type:** Thoracic lung |
| Tian et al., 2023  China | Impact of “information platform + self-care model” on health status | **Design:** RCT  **Setting:** Tertiary specialized women’s and children’s hospital | **n =** 55 patients (28 intervention and 27 control)  **Surgery type:** Vaginal natural orifice transluminal endoscopic |
| Tseng, Shyu, Liang, & Tsai, 2016  Taiwan | Assess interdisciplinary intervention on depressive symptoms | **Design:** Secondary analysis of a RCT  **Setting:** Medical centre | **n =** 153 patients (76 intervention and 77 control)  **n =** 152 caregivers (76 intervention and 76 control)  **Surgery type:** Hip arthroplasty or internal fixation of hip fracture |
| Tseng et al., 2021  Taiwan | Examine family-centered care model on patient health outcomes and caregiver competence and self-efficacy | **Design:** RCT  **Setting:** Medical centre | **n =** 152 patients (76 intervention and 76 control)  **Surgery type:** Hip arthroplasty or internal fixation |
| Wang, Hua, Liu, Liu, & Liang, 2023  China | Investigate nurse-led transitional care programme on discharge readiness, self-care ability, transitional care quality and quality of life | **Design:** Retrospective cohort  **Setting:** Hepatobiliary surgery wards at one tertiary medical centre | **n =** 706 patients (255 intervention and 451 control)  **Surgery type:** Biliary |
| Weintraub et al., 2018  USA | Describe IT enabled care management on readmissions and secondary prevention | **Design:** Observational, using a time-series approach  **Setting:** Hospital^c^ | **n =** 1827 patients (716 intervention and 1111 control)  **Surgery type:** Coronary artery bypass graft |
| Xu, Zhao, Bai, & Li, 2021  China | Evaluate implementation of care transition pathway on care transition quality | **Design:** Quasi-experimental; nonequivalent control group design (posttest only)  **Setting:** Orthopaedic department of a tertiary care hospital | **n =** 96 patients (45 intervention and 51 control)  **Surgery type:** Joint (hip or knee) replacement |
| Yang, Xu, Miao, Geng, & Geng, 2023  China | Investigate transition care programs on activities of daily living, pain levels, social support levels and fracture recurrence | **Design:** Quasi-experimental; time series with control group (posttest only) **Setting:** Public teaching hospital | **n =** 160 patients (80 intervention and 80 control)  **Surgery type:** Percutaneous vertebroplasty |
| (Zhang et al., 2020; Zhang et al., 2021)  China | Evaluate hospital-family holistic care model on patient health outcomes and caregiver outcomes | **Design:** RCT  **Settings:** Medical centres of three large general hospitals | **n =** 119 patients (60 intervention and 59 control)  **n =** 125 caregivers (62 intervention and 63 control)  **Surgery type:** Permanent colostomy |
| Zhou et al., 2020  China | Evaluate WeChat-based multimodal nursing program on early rehabilitation | **Design:** RCT  **Setting:** Surgical breast cancer department of a general hospital | **n =** 111 patients (56 intervention and 55 control)  **Surgery type:** Breast: modified radical mastectomy, total mastectomy, breast-conserving |
| Zuckerman et al., 2020  USA | Create quality improvement tool to reduce length of stay and readmission | **Design:** Prospective cohort  **Settings:** Eight institutions that were all existing members of the Quality Outcomes Database (a national registry that monitors spinal surgical care) | **n =** 209 patients  **Surgery type:** Lumbar fusion |

RCT, randomised controlled trial; USA, United States of America

^a^‘Control’ means unmatched cohort

^b^Only 830 patients were used for the cost analysis. Note: the cost benefit analysis had n = 490 cases and n = 415 controls; 40% of eligible patients were offered the TCP (determined by convenience) and then compared to controls matched by age, sex and operation type

^c^No further information reported

Ahmadi, N., Mbuagbaw, L., Finley, C., Agzarian, J., Hanna, W. C., & Shargall, Y. (2021). Impact of the integrated comprehensive care program post-thoracic surgery: A propensity score–matched study. *The Journal of Thoracic and Cardiovascular Surgery, 162*(1), 321-330.e321. doi:https://doi.org/10.1016/j.jtcvs.2020.05.095

Aicher, B. O., Hanlon, E., Rosenberger, S., Toursavadkohi, S., & Crawford, R. S. (2019). Reduced length of stay and 30-day readmission rate on an inpatient vascular surgery service. *Journal of Vascular Nursing, 37*(2), 78-85. doi:https://doi.org/10.1016/j.jvn.2018.11.004

Akbari, M., & Celik, S. S. (2018). The effects of discharge training and postdischarge counseling on quality of life after coronary artery bypass graft surgery. *Nursing and Midwifery Studies, 7*(3), 105-110.

Borregaard, B., Dahl, J. S., Riber, L. P. S., Ekholm, O., Sibilitz, K. L., Weiss, M., . . . Møller, J. E. (2019). Effect of early, individualised and intensified follow-up after open heart valve surgery on unplanned cardiac hospital readmissions and all-cause mortality. *International Journal of Cardiology, 289*, 30-36.

Coskun, S., & Duygulu, S. (2022). The effects of Nurse Led Transitional Care Model on elderly patients undergoing open heart surgery: a randomized controlled trial. *European Journal of Cardiovascular Nursing, 21*(1), 46-55.

Du, R. Y., Shelton, G., Ledet, C. R., Mills, W. L., Neal-Herman, L., Horstman, M., . . . Naik, A. D. (2021). Implementation and feasibility of the re-engineered discharge for surgery (RED-S) intervention: A pilot study. *Journal for Healthcare Quality: official publication of the National Association for Healthcare Quality 43*(2), 92.

Fisher, A. V., Campbell-Flohr, S. A., Sell, L., Osterhaus, E., Acher, A. W., Leahy-Gross, K., . . . Abbott, D. E. (2018). Adaptation and implementation of a transitional care protocol for patients undergoing complex abdominal surgery. *The Joint Commission Journal on Quality and Patient Safety, 44*(12), 741-750.

Fitz, S., Diegel-Vacek, L., & Mahoney, E. (2020). A performance improvement initiative for implementing an evidence-based discharge bundle for lung transplant recipients. *Progress in Transplantation, 30*(3), 281-285.

Grahn, S. W., Lowry, A. C., Osborne, M. C., Melton, G. B., Gaertner, W. B., Vogler, S. A., . . . Kwaan, M. R. (2019). System-wide improvement for transitions after ileostomy surgery: can intensive monitoring of protocol compliance decrease readmissions? A randomized trial. *Diseases of the Colon & Rectum, 62*(3), 363-370.

Hu, R., Gu, B., Tan, Q., Xiao, K., Li, X., Cao, X., . . . Jiang, X. (2020). The effects of a transitional care program on discharge readiness, transitional care quality, health services utilization and satisfaction among Chinese kidney transplant recipients: A randomized controlled trial. *International Journal of Nursing Studies, 110*, 103700.

Iseler, J., Fox, J., & Wierenga, K. (2018). Performance improvement to decrease readmission rates for patients with a left ventricular assist device. *Progress in Transplantation, 28*(2), 184-188.

Koçan, S., & Gürsoy, A. (2023). Outcomes of breast care nurse training and follow-up: body image, anxiety, and quality of life. *Journal of Education and Research in Nursing 20*(1), 52-59.

Koeckert, M. S., Ursomanno, P. A., Williams, M. R., Querijero, M., Zias, E. A., Loulmet, D. F., . . . Galloway, A. C. (2017). Reengineering valve patients' postdischarge management for adapting to bundled payment models. *The Journal of Thoracic and Cardiovascular Surgery, 154*(1), 190-198.

Lee, J. (2017). Transitional care intervention: A readmission solution. *Nursing Management, 48*(3), 32-39.

Lee, J. H., Kang, S.-M., Kim, Y. A., & Chu, S. H. (2021). Clinical outcomes of a nurse-led post-discharge education program for heart-transplant recipients: a retrospective cohort study. *Applied Nursing Research, 59*, 151427.

Li, L., Ma, Z., & Wang, W. (2020). Influence of transitional care on the self-care ability of kidney transplant recipients after discharge. *Annals of Palliative Medicine, 9*(4), 1958964-1951964.

Liu, J., Gormley, N., Dasenbrock, H. H., Aglio, L. S., Smith, T. R., Gormley, W. B., & Robertson, F. C. (2019). Cost-benefit analysis of transitional care in neurosurgery. *Neurosurgery, 85*(5), 672-679.

Mitchell, K. (2022). Impact of Reengineered Discharge Toolkit on Patients Undergoing Total Joint Surgeries. *Rehabilitation Nursing Journal, 47*(4), 121-128.

Pelt, C. E., Gililland, J. M., Erickson, J. A., Trimble, D. E., Anderson, M. B., & Peters, C. L. (2018). Improving Value in Total Joint Arthroplasty: A Comprehensive Patient Education and Management Program Decreases Discharge to Post-Acute Care Facilities and Post-Operative Complications. *The Journal of Arthroplasty, 33*(1), 14-18. doi:10.1016/j.arth.2017.08.003

Robertson, F. C., Logsdon, J. L., Dasenbrock, H. H., Yan, S. C., Raftery, S. M., Smith, T. R., & Gormley, W. B. (2018). Transitional care services: a quality and safety process improvement program in neurosurgery. *Journal of Neurosurgery, 128*(5), 1570-1577. doi:10.3171/2017.2.Jns161770

Shargall, Y., Hanna, W. C., Schneider, L., Schieman, C., Finley, C. J., Tran, A., . . . Blackhouse, G. (2016). *The integrated comprehensive care program: a novel home care initiative after major thoracic surgery.* Paper presented at the Seminars in thoracic and cardiovascular surgery.

Tian, T., Guan, M.-J., Liu, L.-J., Su, X.-Q., Wang, H., & He, L. (2023). Study on the Efficacy of “Information Platform+ Self-Care Model” on the Health Status of Discharged Patients Following Vaginal Natural Orifice Transluminal Endoscopic Surgery. *International Journal of Women's Health*, 1185-1195.

Tseng, M.-Y., Yang, C.-T., Liang, J., Huang, H.-L., Kuo, L.-M., Wu, C.-C., . . . Lee, P.-C. (2021). A family care model for older persons with hip-fracture and cognitive impairment: A randomized controlled trial. *International Journal of Nursing Studies, 120*, 103995.

Tseng, M. Y., Shyu, Y. I. L., Liang, J., & Tsai, W. C. (2016). Interdisciplinary intervention reduced the risk of being persistently depressive among older patients with hip fracture. *Geriatrics & gerontology international, 16*(10), 1145-1152.

Wang, M., Hua, J., Liu, Y., Liu, T., & Liang, H. (2023). Application of a nurse‐led transitional care programme for patients discharged with T‐tubes after biliary surgery. *Nursing Open*(10), 4570-4577.

Weintraub, W. S., Elliott, D., Fanari, Z., Ostertag-Stretch, J., Muther, A., Lynahan, M., . . . Anderson, S. (2018). The impact of care management information technology model on quality of care after coronary artery bypass surgery:“Bridging the Divides”. *Cardiovascular Revascularization Medicine, 19*(1), 106-111.

Xu, Y.-p., Zhao, P.-y., Bai, Y.-t., & Li, S. (2021). The effect of care transition pathway implementation on patients undergoing joint replacement during the COVID-19 pandemic: a quasi-experimental study from a tertiary care hospital orthopedic department in Beijing, China. *Journal of Orthopaedic Surgery and Research, 16*(1), 1-5.

Yang, W., Xu, H., Miao, W., Geng, Z., & Geng, G. (2023). Effects of transitional care based on the social support theory for older patients with osteoporotic vertebral compression fractures: A quasi‐experimental trial. *Australasian Journal on Ageing, 42*(1), 185-194.

Zhang, X., Gao, R., Lin, J. L., Chen, N., Lin, Q., Huang, G. F., . . . Li, H. (2020). Effects of hospital‐family holistic care model on the health outcome of patients with permanent enterostomy based on the theory of ‘Timing It Right’. *Journal of Clinical Nursing, 29*(13-14), 2196-2208.

Zhang, X., Lin, J. L., Gao, R., Chen, N., Huang, G. F., Wang, L., . . . Chen, X. H. (2021). Application of the hospital‐family holistic care model in caregivers of patients with permanent enterostomy: A randomized controlled trial. *Journal of Advanced Nursing, 77*(4), 2033-2049.

Zhou, K., Wang, W., Zhao, W., Li, L., Zhang, M., Guo, P., . . . Li, J. (2020). Benefits of a WeChat-based multimodal nursing program on early rehabilitation in postoperative women with breast cancer: a clinical randomized controlled trial. *International Journal of Nursing Studies, 106*, 103565.

Zuckerman, S. L., Devin, C. J., Rossi, V., Chotai, S., Dyer, E. H., Knightly, J. J., . . . Glassman, S. D. (2020). The Institute for Healthcare Improvement–NeuroPoint Alliance collaboration to decrease length of stay and readmission after lumbar spine fusion: using national registries to design quality improvement protocols. *Journal of Neurosurgery: Spine, 33*(6), 812-821.
