# Supplementary material for: Surgical transitional care interventions and their outcomes: a scoping review
Source: Int J Nurs Stud Adv. 2025 Apr 8;8:100328. doi: 10.1016/j.ijnsa.2025.100328 (PMC12136900; doi:10.1016/j.ijnsa.2025.100328)
Supplement: Supplementary file 4 [file mmc4.docx]

**Supplementary File 4.** Intervention characteristics

| Author and year | Core (required) and discretionary components | Bundle foundation |
| --- | --- | --- |
| Ahmadi et al., 2021 | All core | - Literature review |
| Aicher, Hanlon, Rosenberger, Toursavadkohi, & Crawford, 2019 | All core | - Review of previously published tools used to develop and modify telephone questionnaire |
| Akbari & Celik, 2018 | All core | - Literature review - Stakeholder input (education booklet tested with stakeholders): nursing experts, heart surgeons, nursing and midwifery faculty members, patients |
| Borregaard et al., 2019 | All core | - Literature review |
| Coskun & Duygulu, 2022 | All core | - Literature review - Transitional care model application protocol |
| Du et al., 2021 | All core | - Adaptation of the Project Re-Engineered Discharge for surgical patients - Stakeholder input: surgical staff members (including frontline clinicians and supervisory staff) |
| Fisher et al., 2018 | All core | - Previous transitional care intervention protocols - Literature review - Stakeholder input: surgeons, inpatient and outpatient nurses, case managers, pharmacists, hospital administrators, and patients |
| Fitz, Diegel-Vacek, & Mahoney, 2020 | All core | - Literature review - Havelock and Zlotolow’s Theory of Planned Change - Motivational interviews with interdisciplinary lung transplant team - Stakeholder input: nurse practitioners |
| Grahn et al., 2019 | All core | - Revise system-wide patient education brochure - Stakeholders input: surgeons, nurses, wound ostomy continence nurses, and patient education specialists - Literature review |
| Hu et al., 2020 | All core | - Literature review and review of related guidelines - Stakeholder input: kidney transplant specialists, kidney transplant nursing specialists, and chronic disease management specialists |
| Iseler, Fox, & Wierenga, 2018 | All core | - Literature review |
| Koçan & Gürsoy, 2023 | All core | - Clinical nursing experience of researcher - Resources, such as the ‘Breast cancer surgery patient handbook’ - Literature review - Stakeholder input (training book): general surgeons, psychiatrist, medical-surgical nurses, a faculty member and a nurse in charge of a general surgery clinic |
| Koeckert et al., 2017 | All core | - Stakeholder input: cardiac service line team |
| Lee, 2017 | Most core; caregiver involvement dependent on caregiver level of comprehension | - Middle-range transitions theory - Literature review |
| Lee, Kang, Kim, & Chu, 2021 | All core | - Literature review |
| Li, Ma, & Wang, 2020 | All core | - Literature review |
| Liu et al., 2019; Robertson et al., 2018 | All core | - Literature review |
| Pelt et al., 2018 | All core | - Stakeholder input (interviews and focus group meetings at the study hospital): patients and providers |
| Shargall et al., 2016 | All core | - Not explicit |
| Tian et al., 2023 | All core | - Evidence-based method - Literature review - Expert consultation: unclear who experts were |
| Tseng, Shyu, Liang, & Tsai, 2016 | All core | - Literature review |
| Tseng et al., 2021 | All core | - Literature review - “Family-centred model” as per research team’s prior studies |
| Wang, Hua, Liu, Liu, & Liang, 2023) | All core | - Literature review - Relevant guidelines - Expert consultation: unclear who experts were |
| Weintraub et al., 2018 | All core | - Literature review |
| Xu, Zhao, Bai, & Li, 2021 | All core | - Stakeholder input: multidisciplinary team |
| Yang, Xu, Miao, Geng, & Geng, 2023 | All core | - Literature review - Social support theory |
| Zhang et al., 2020; Zhang et al., 2021 | All core | - Theory - Literature review - Development and treatment characteristics of rectal cancer - Stakeholder input: specialist doctor opinion, patients with colorectal cancer and caregivers |
| Zhou et al., 2020 | All core | - Literature review - Theoretical framework - Stakeholder input: doctor and nurse from the surgical breast cancer department, and postgraduate trainee majoring in breast cancer care |
| Zuckerman et al., 2020 | All core | - Literature review - Institute for Healthcare Improvement resources - Stakeholder input: surgeons, nurses, midlevel providers, and clinic staff |

**References**

Ahmadi, N., Mbuagbaw, L., Finley, C., Agzarian, J., Hanna, W. C., & Shargall, Y. (2021). Impact of the integrated comprehensive care program post-thoracic surgery: A propensity score–matched study. *The Journal of Thoracic and Cardiovascular Surgery, 162*(1), 321-330.e321. doi:https://doi.org/10.1016/j.jtcvs.2020.05.095

Aicher, B. O., Hanlon, E., Rosenberger, S., Toursavadkohi, S., & Crawford, R. S. (2019). Reduced length of stay and 30-day readmission rate on an inpatient vascular surgery service. *Journal of Vascular Nursing, 37*(2), 78-85. doi:https://doi.org/10.1016/j.jvn.2018.11.004

Akbari, M., & Celik, S. S. (2018). The effects of discharge training and postdischarge counseling on quality of life after coronary artery bypass graft surgery. *Nursing and Midwifery Studies, 7*(3), 105-110.

Borregaard, B., Dahl, J. S., Riber, L. P. S., Ekholm, O., Sibilitz, K. L., Weiss, M., . . . Møller, J. E. (2019). Effect of early, individualised and intensified follow-up after open heart valve surgery on unplanned cardiac hospital readmissions and all-cause mortality. *International Journal of Cardiology, 289*, 30-36.

Coskun, S., & Duygulu, S. (2022). The effects of Nurse Led Transitional Care Model on elderly patients undergoing open heart surgery: a randomized controlled trial. *European Journal of Cardiovascular Nursing, 21*(1), 46-55.

Du, R. Y., Shelton, G., Ledet, C. R., Mills, W. L., Neal-Herman, L., Horstman, M., . . . Naik, A. D. (2021). Implementation and feasibility of the re-engineered discharge for surgery (RED-S) intervention: A pilot study. *Journal for Healthcare Quality: official publication of the National Association for Healthcare Quality 43*(2), 92.

Fisher, A. V., Campbell-Flohr, S. A., Sell, L., Osterhaus, E., Acher, A. W., Leahy-Gross, K., . . . Abbott, D. E. (2018). Adaptation and implementation of a transitional care protocol for patients undergoing complex abdominal surgery. *The Joint Commission Journal on Quality and Patient Safety, 44*(12), 741-750.

Fitz, S., Diegel-Vacek, L., & Mahoney, E. (2020). A performance improvement initiative for implementing an evidence-based discharge bundle for lung transplant recipients. *Progress in Transplantation, 30*(3), 281-285.

Grahn, S. W., Lowry, A. C., Osborne, M. C., Melton, G. B., Gaertner, W. B., Vogler, S. A., . . . Kwaan, M. R. (2019). System-wide improvement for transitions after ileostomy surgery: can intensive monitoring of protocol compliance decrease readmissions? A randomized trial. *Diseases of the Colon & Rectum, 62*(3), 363-370.

Hu, R., Gu, B., Tan, Q., Xiao, K., Li, X., Cao, X., . . . Jiang, X. (2020). The effects of a transitional care program on discharge readiness, transitional care quality, health services utilization and satisfaction among Chinese kidney transplant recipients: A randomized controlled trial. *International Journal of Nursing Studies, 110*, 103700.

Iseler, J., Fox, J., & Wierenga, K. (2018). Performance improvement to decrease readmission rates for patients with a left ventricular assist device. *Progress in Transplantation, 28*(2), 184-188.

Koçan, S., & Gürsoy, A. (2023). Outcomes of breast care nurse training and follow-up: body image, anxiety, and quality of life. *Journal of Education and Research in Nursing 20*(1), 52-59.

Koeckert, M. S., Ursomanno, P. A., Williams, M. R., Querijero, M., Zias, E. A., Loulmet, D. F., . . . Galloway, A. C. (2017). Reengineering valve patients' postdischarge management for adapting to bundled payment models. *The Journal of Thoracic and Cardiovascular Surgery, 154*(1), 190-198.

Lee, J. (2017). Transitional care intervention: A readmission solution. *Nursing Management, 48*(3), 32-39.

Lee, J. H., Kang, S.-M., Kim, Y. A., & Chu, S. H. (2021). Clinical outcomes of a nurse-led post-discharge education program for heart-transplant recipients: a retrospective cohort study. *Applied Nursing Research, 59*, 151427.

Li, L., Ma, Z., & Wang, W. (2020). Influence of transitional care on the self-care ability of kidney transplant recipients after discharge. *Annals of Palliative Medicine, 9*(4), 1958964-1951964.

Liu, J., Gormley, N., Dasenbrock, H. H., Aglio, L. S., Smith, T. R., Gormley, W. B., & Robertson, F. C. (2019). Cost-benefit analysis of transitional care in neurosurgery. *Neurosurgery, 85*(5), 672-679.

Pelt, C. E., Gililland, J. M., Erickson, J. A., Trimble, D. E., Anderson, M. B., & Peters, C. L. (2018). Improving Value in Total Joint Arthroplasty: A Comprehensive Patient Education and Management Program Decreases Discharge to Post-Acute Care Facilities and Post-Operative Complications. *The Journal of Arthroplasty, 33*(1), 14-18. doi:10.1016/j.arth.2017.08.003

Robertson, F. C., Logsdon, J. L., Dasenbrock, H. H., Yan, S. C., Raftery, S. M., Smith, T. R., & Gormley, W. B. (2018). Transitional care services: a quality and safety process improvement program in neurosurgery. *Journal of Neurosurgery, 128*(5), 1570-1577. doi:10.3171/2017.2.Jns161770

Shargall, Y., Hanna, W. C., Schneider, L., Schieman, C., Finley, C. J., Tran, A., . . . Blackhouse, G. (2016). *The integrated comprehensive care program: a novel home care initiative after major thoracic surgery.* Paper presented at the Seminars in thoracic and cardiovascular surgery.

Tian, T., Guan, M.-J., Liu, L.-J., Su, X.-Q., Wang, H., & He, L. (2023). Study on the Efficacy of “Information Platform+ Self-Care Model” on the Health Status of Discharged Patients Following Vaginal Natural Orifice Transluminal Endoscopic Surgery. *International Journal of Women's Health*, 1185-1195.

Tseng, M.-Y., Yang, C.-T., Liang, J., Huang, H.-L., Kuo, L.-M., Wu, C.-C., . . . Lee, P.-C. (2021). A family care model for older persons with hip-fracture and cognitive impairment: A randomized controlled trial. *International Journal of Nursing Studies, 120*, 103995.

Tseng, M. Y., Shyu, Y. I. L., Liang, J., & Tsai, W. C. (2016). Interdisciplinary intervention reduced the risk of being persistently depressive among older patients with hip fracture. *Geriatrics & gerontology international, 16*(10), 1145-1152.

Wang, M., Hua, J., Liu, Y., Liu, T., & Liang, H. (2023). Application of a nurse‐led transitional care programme for patients discharged with T‐tubes after biliary surgery. *Nursing Open*(10), 4570-4577.

Weintraub, W. S., Elliott, D., Fanari, Z., Ostertag-Stretch, J., Muther, A., Lynahan, M., . . . Anderson, S. (2018). The impact of care management information technology model on quality of care after coronary artery bypass surgery:“Bridging the Divides”. *Cardiovascular Revascularization Medicine, 19*(1), 106-111.

Xu, Y.-p., Zhao, P.-y., Bai, Y.-t., & Li, S. (2021). The effect of care transition pathway implementation on patients undergoing joint replacement during the COVID-19 pandemic: a quasi-experimental study from a tertiary care hospital orthopedic department in Beijing, China. *Journal of Orthopaedic Surgery and Research, 16*(1), 1-5.

Yang, W., Xu, H., Miao, W., Geng, Z., & Geng, G. (2023). Effects of transitional care based on the social support theory for older patients with osteoporotic vertebral compression fractures: A quasi‐experimental trial. *Australasian Journal on Ageing, 42*(1), 185-194.

Zhang, X., Gao, R., Lin, J. L., Chen, N., Lin, Q., Huang, G. F., . . . Li, H. (2020). Effects of hospital‐family holistic care model on the health outcome of patients with permanent enterostomy based on the theory of ‘Timing It Right’. *Journal of Clinical Nursing, 29*(13-14), 2196-2208.

Zhang, X., Lin, J. L., Gao, R., Chen, N., Huang, G. F., Wang, L., . . . Chen, X. H. (2021). Application of the hospital‐family holistic care model in caregivers of patients with permanent enterostomy: A randomized controlled trial. *Journal of Advanced Nursing, 77*(4), 2033-2049.

Zhou, K., Wang, W., Zhao, W., Li, L., Zhang, M., Guo, P., . . . Li, J. (2020). Benefits of a WeChat-based multimodal nursing program on early rehabilitation in postoperative women with breast cancer: a clinical randomized controlled trial. *International Journal of Nursing Studies, 106*, 103565.

Zuckerman, S. L., Devin, C. J., Rossi, V., Chotai, S., Dyer, E. H., Knightly, J. J., . . . Glassman, S. D. (2020). The Institute for Healthcare Improvement–NeuroPoint Alliance collaboration to decrease length of stay and readmission after lumbar spine fusion: using national registries to design quality improvement protocols. *Journal of Neurosurgery: Spine, 33*(6), 812-821.
